# Sex-specific effects of early-life adversity on adult fitness in a wild mammal

**DOI:** 10.1098/rspb.2025.0192

**Published:** 2025-03-26

**Authors:** Elizabeth D. Drake, Sanjana Ravindran, Xavier Bal, Josephine M. Pemberton, Jill G. Pilkington, Daniel H. Nussey, Hannah Froy

**Affiliations:** ^1^ Institute of Ecology and Evolution, The University of Edinburgh School of Biological Sciences, Edinburgh, Edinburgh, UK

**Keywords:** early-life environment, carry-over effects, silver spoon effects, lifetime breeding success, longevity, Soay sheep (*Ovis aries*)

## Abstract

Early-life adversity influences adult fitness across vertebrate species. In polygynous systems with intense intrasexual competition, males may be more sensitive to conditions experienced during development. However, the importance of different aspects of the early-life environment and how their effects differ between the sexes remains poorly understood. Here, we used a long-term study of wild Soay sheep to characterize the early-life environment in terms of weather, infection risk, resource competition and maternal investment, and test the hypothesis that males are more vulnerable to early adversity. Birth weight, reflective of maternal investment and conditions during gestation, positively predicted lifetime breeding success in both sexes, suggesting a classic ‘silver spoon’ effect, though the effects were stronger in males. Males that experienced increased resource competition in their first year had lower lifetime breeding success, suggesting lasting negative consequences of nutritional stress, but there was no association in females. By contrast, challenging weather in the first winter of life was associated with stronger viability selection, with males surviving these harsh conditions having higher adult fitness. Our findings further evidence the important long-term fitness consequences of early-life adversity in wild vertebrates, demonstrating distinct aspects of the early environment may shape fitness in different and sex-specific ways.

## 1. Introduction

Development is particularly sensitive to the environment, and the conditions experienced during this life stage can have lasting impacts on physiological function, demographic rates and ultimately lifetime fitness [1,2]. Thus, early-life environment can have important consequences for ecological and evolutionary processes, shaping population dynamics and life history evolution [2–4]. Numerous studies show that favourable conditions during early life can have positive consequences for adult body size, reproduction and survival, coined ‘silver spoon’ effects [5–8]. However, challenging early-life conditions may also be associated with stronger viability selection, which may result in the selective disappearance of weaker individuals from a cohort [6,9–13]. This ‘invisible fraction’ may mean the surviving members of cohorts experiencing strong early viability selection have higher mean adult fitness compared with cohorts experiencing benign conditions [11,13–16]. Additionally, many human and primate studies have developed composite, cumulative measures of early-life adversity and shown high adversity scores predict lower adult health and fitness [17–19]. However, natural populations experience complex, fluctuating environments involving diverse challenges including resource competition, harsh weather, constraints on parental investment and parasite infection. These challenges may affect entire cohorts, or they may vary at the level of the individual, and could impact development and fitness via different processes and to different degrees. Yet most studies linking early-life environment and fitness to date have used composite measures of adversity or a small number of environmental variables [20–22]. In this study, we provide a rare test of how different aspects of early-life adversity shape different fitness traits in a wild vertebrate population living in a highly variable environment.

In polygynous systems, the effects of early-life conditions on later-life fitness are expected to differ between the sexes due to the different selection pressures on males and females [2,23,24]. Greater sexual competition among males often leads to more rapid growth rates, larger body size and the development of costly secondary sexual characteristics in such systems. Males may therefore have greater nutritional and energy requirements and increased sensitivity to environmental adversity during gestation and neonatal periods [25]. This may lead to sex-biases in juvenile mortality in sexually dimorphic species, which could impose stronger viability selection on males [4]. Furthermore, polygynous males exhibit highly skewed and more variable reproductive success. Coupled with their heightened sensitivity to early conditions, this may increase the likelihood of males showing long-lasting silver spoon effects on adult fitness [15,26]. In support of this, the negative survival consequences of orphaning were stronger in wild male chimpanzees and red deer [24,27], as were those of lower early-life social connectivity in bottlenose dolphins [24,28]. Similarly, sex-dependent effects of birth weight, population density and climate in the year of birth have been reported in moose and red deer [29,30]. However, to date, only a handful of studies have tested for differential impacts on both reproduction and survival across the entire lifespan in polygynous systems. Here, we use individual-based data from a long-term study of a highly polygynous mammal to test the prediction that early-life adversity has a stronger effect on adult fitness in males than females.

The long-term study of Soay sheep on St Kilda offers detailed, longitudinal data on morphology, life history and demography collected across the lifetimes of thousands of individuals over four decades [31]. The mating system is highly polygynous: adult males are 60% heavier and face strong intra-sexual competition for access to receptive females during the mating season [31,32]. Females give birth to singleton or twin lambs in the spring, whereas male annual and lifetime reproductive success are highly skewed, with most males gaining no or few paternities (electronic supplementary material, figure S1). Winter mortality is also higher and more sensitive to resource competition in males [32,33]. The early-life environmental factors impacting first year survival are well understood: lambs that are born lighter, into larger litters and suffer early maternal loss are less likely to survive their first winter, as are those born into cohorts that experience higher population densities, increased parasite exposure and wetter and windier winter weather conditions during the first year of life [31,34–36].

In this study, we linked this broad suite of early environmental challenges, measured at both the individual and cohort levels, to adult fitness in both sexes. We predicted that our individual-level measures of early adversity (low birth weight, high natal litter size, maternal loss) would show classic silver spoon effects, being negatively associated with adult fitness components. Associations with the cohort-level predictors (high population density, high parasite exposure, harsh winter weather) may be more complex, depending on both the strength of the resulting viability selection and the potential for long-lasting impacts on the physiology or behaviour of the survivors. We predicted that the long-term costs of nutritional stress and mounting an immune response would have negative impacts on adult fitness for those that experienced high densities and parasite exposure. Harsh winter weather, however, may have shorter term consequences in terms of stronger viability selection and the removal of weak individuals from the cohort, resulting in positive associations with adult performance. We expected male adult fitness to be more strongly impacted in both scenarios. In line with our predictions, we demonstrate that different aspects of the early environment shape adult fitness in different ways, through different processes and via different components of fitness, highlighting the complexity of sex-dependent early-life effects.

## 2. Methods

### (a) Study system and data

Since 1985, the Soay sheep resident to the Village Bay area of Hirta, St Kilda, Scotland have been the subject of an individual-based study [31]. During the mating season in October–November, males compete to gain access to oestrous ewes and sequentially mate with multiple females while forming consort pairs. After an approximately five-month gestation period, lambs are born in April–May and more than 90% of lambs born in the study area are caught within a few weeks of birth. At capture, lambs are uniquely marked with ear tags, tissue sampled and weighed. Each August, 50–70% of the population are caught and weighed, and faecal samples are taken. Regular censuses mean that the fate of individuals is known with a high degree of certainty. Mortality peaks in late winter/early spring, and around 80% of deceased sheep are found [31].

In this study, we used data collected between 1985 and 2022. To test for persistent effects of early-life adversity on fitness, we developed a list of candidate early-life metrics and tested whether they independently predicted first year survival. We then tested whether they were associated with components of lifetime fitness (see below) in individuals that survived beyond their first year of life, and whether those associations differed between the sexes.

### (b) Early-life measures

#### (i) Individual-level predictors

#### Birth weight

Residuals from a generalized additive model of weight in kilograms for individuals weighed within the first 7 days of life, with a smoothing term for age at capture in days (package *mcgv* [37]). These residuals do not account for uncertainty in this relationship, but it was important to account for variation in age at capture during this rapid growth phase (electronic supplementary material, figure S2) and we do not expect this to introduce bias into our analyses. At least 60% of individuals in each cohort were captured and weighed within a week of birth, except in 2001 and 2020 where logistical challenges resulted in a high proportion of missing data (foot and mouth outbreak and COVID-19 pandemic, respectively). See §2d below for how these cohorts were handled, along with individuals that were not caught within the first week of life. Birth weight captures elements of the gestational environment, such as the weather conditions and population density experienced by mothers when the lamb is *in utero* [38], as well as maternal home range quality and investment [34]. It is positively associated with subsequent growth rates and first winter survival in this system [34,36].

#### Natal litter size

Coded as singleton or twin. Around 18% of Soay sheep births are of twins, and triplets are very rare (seven sets recorded, grouped with twins in this analysis). Twins are lighter than singletons at birth and in August and have reduced first year survival [36].

#### Maternal loss

A binary trait indicating whether an individual lost their mother in their first year of life (i.e. whether mother died before 1 May in the year following birth or survived). For a very small number of cases, the mother’s month of death was not known with certainty. In these cases, we assumed the mother had died over winter in the recorded year of death (<1% of records) or, if year of death was unknown, we assumed the mother had died over winter in the year she was last seen in a census (<1%).

#### (ii) Cohort-level predictors

#### Parasite exposure in year of birth

Mean faecal egg count (FEC) across the cohort of lambs in August. The number of strongyle nematode eggs was counted as a proxy of parasite burden in each faecal sample collected from sheep at August capture using a modified McMaster technique: faecal matter was mixed with NaCl, broken down, filtered and run into a counting chamber where the number of strongyle eggs present was counted to estimate the number of eggs per gram of wet weight of faeces [39,40]. As not every lamb is caught and faecal sampled in August, roughly 68% of the dataset was missing a value for individual faecal egg count. We therefore used the average faecal egg count across a cohort of lambs as an indicator of parasite exposure (following [41]). Two extreme outlying individual values (>10 000) were removed before calculating the mean for each cohort. Faecal egg counts were unavailable for the earliest cohorts (1985−1987) and for >90% lambs in 2002, and counts were performed using a different method in recent years (2019−2021). See §2d below for how these missing data were handled. Strongyle gastrointestinal parasites are highly prevalent in this population and faecal egg count predicts summer weight and overwinter mortality in lambs [42–44]. As lambs are naïve to gut parasites at birth, the mean density of parasite eggs found in lamb faecal samples is considered a good indicator of the general parasite exposure risk in a given year [39].

#### First winter North Atlantic Oscillation

Hurrell North Atlantic Oscillation Index for the winter after birth (December–March). North Atlantic Oscillation (NAO) is a measure of atmospheric pressure across the North Atlantic region, based on the relative difference in pressure between stations in Portugal and Iceland. Positive NAO values indicate mild, stormy and wet winter conditions in Northern Europe and negative values indicate cold, calm and dry winter weather. Positive winter NAO values predict increased winter mortality in this population [33]. We used station-based estimates of NAO from the NCAR Climate Data Guide (https://climatedataguide.ucar.edu/climate-data/hurrell-north-atlantic-oscillation-nao-index-station-based).

#### Population size in year of birth

Total number of individuals alive resident in the Village Bay area on 1 October following birth, estimated from birth and death dates and census records. High population sizes are associated with increased subsequent winter mortality, particularly in lambs, presumably through increased resource competition [33,44].

### (c) Fitness components

First year survival was defined as survival to 1 May in the year following birth (0: died before 1 May in year following birth; 1: survived to 1 May in year following birth). A small proportion of individuals were assigned assuming late winter/early spring mortality in the recorded year of death when death month was unknown (<5%) or based on the last time they were seen in a census (<1%). Our analyses of first year survival included data from all individuals born between 1985 and 2021 who survived to 1 October in their year of birth, where sex, birth year, maternal identity and the fate of the mother were known (*n* = 5073).

Our analyses of adult fitness traits were restricted to individuals who survived beyond their first year of life, to ensure any associations with early-life adversity were independent of first year survival. Reproductive performance in both sexes was calculated based on a multigenerational genetic pedigree, inferred using a subset of 431 unlinked Single Nucleotide Polymorphisms (SNPs) derived from the Illumina Ovine 50K SNP array using the R package Sequoia [45,46]. For a small number of cases where SNP genotype information was not available, parentage assignments were made using field observations (for females; 11.2%) or from microsatellite data (for males; 3.0%) [47].

Lifetime breeding success (LBS) was defined as the number of offspring born to or sired by an individual over their lifespan. This ranged between 0 and 20 for females and 0 and 94 for males. To understand whether differences in LBS were driven by differences in survival or reproduction, we decomposed LBS into its constituent components of longevity and annual reproductive performance. Longevity was the age of an individual at death, ranging from 1 to 15 for females and 1 to 11 for males. Our analyses of LBS and longevity were restricted to individuals born 1985−2017 that had completed their natural lifespans and for whom year of death was known (*n* = 1721; 1034 females and 687 males). Where information on the month of death was missing, late winter/early spring mortality was assumed when assigning longevity (*n* = 288) unless an individual was seen in a census later that year (*n* = 20).

Analyses of annual reproductive performance measures included individuals that were still alive. We used all observations from individuals born 1985−2021 where age ≥ 1. Breeding probability was a binary trait indicating whether an individual did (1) or did not (0) give birth to or sire an offspring in each year of their life (*n* = 9572 observations of 2232 individuals). For females that bred in a given year, twinning probability was a binary trait indicating whether the female gave birth to a single lamb (0) or twins (1) (*n* = 5580 observations of 1092 females). For males that bred in a given year, offspring number was the number of live offspring they sired that year, ranging from 1 to 22 (*n* = 884 observations of 378 males).

### (d) Statistical analyses

We tested for associations between our metrics of early-life adversity and our fitness components using generalized linear mixed effects models (GLMMs). First year survival, breeding probability and female twinning probability were modelled with a binomial error distribution and logit link function. Lifetime breeding success, longevity and male offspring number were modelled with a negative binomial error distribution and log link function.

First winter NAO, population size in year of birth, cohort mean faecal egg count and birth weight were included in all models as fixed covariates. Natal litter size and maternal loss were included as two-level fixed factors. Sex was included as a two-level fixed factor in all models that included both males and females to account for differences in average trait values between the sexes. To test for sex-specific effects of early-life adversity, we also included two-way interactions between sex and each of our early-life metrics in these models. As harsh climatic conditions could exacerbate the effects of resource competition or poor body condition, we also tested for two-way interactions between first winter NAO and (i) population size, and (ii) birth weight in our model of first year survival.

All models included cohort and maternal identity as random intercept terms to account for shared environmental conditions experienced over the lifetime and non-independence of offspring born to the same mother, respectively. The models of annual fitness components (breeding probability, female twinning probability and male offspring number) also included measurement year and individual identity as random intercept terms. The annual fitness models additionally included a quadratic effect of age in years as a covariate to account for age-related variation in reproductive performance [48]. Interactions between the age terms and sex were included in the model of breeding probability to allow the ageing trajectories to differ between males and females.

All covariates were z-standardized prior to inclusion in the models to aid interpretability (mean = 0 and standard deviation = 1). Interactions that were not statistically significant at the *p* > 0.05 level were sequentially deleted from the model, beginning with the interaction with the highest *p*‐value. All main effects were retained in the model regardless of statistical significance (see electronic supplementary material, table S1 for full models including all interaction terms).

Where data on birth weight or cohort mean faecal egg count were missing (approx. 20% and approx. 15% of lambs, respectively), we used a data imputation approach assuming the data were missing at random to avoid a reduction in sample size [49]. For each of our models, the relevant data subset was used to calculate the mean value for each trait (birth weight or cohort mean faecal egg count), and this was used where data were missing. To check whether this approach impacted our results, we re-ran each of our models excluding the imputed data, and examined whether the results remained the same for birth weight or cohort mean faecal egg count, respectively. Our results remained unchanged (data not shown).

Lastly, we re-ran our final models controlling for mean adult weight to test whether the associations between early-life environment and adult fitness were mediated by adult body weight. Adult weight in kilograms was measured in August. To control for variation in capture age and sex, we took the residuals of a generalized additive model of August weight with a smoothing term for age at capture (in years), a two-level fixed factor for sex, and the interaction between the two, using the R package *mgcv* [37]. We then calculated the mean residual August weight for each individual from all captures where age ≥ 1 and included this as a fixed covariate in our models of adult fitness. We used mean adult weight in the models of LBS and longevity as there was only one observation per individual, meaning it was not possible to include annual measures of weight. We included the same measure of mean adult weight in our models of annual reproductive success as not all sheep are caught every August and complete data on summer weight are therefore not available for every individual.

All statistical analyses were performed using R and RStudio [50,51]. All models were run using the package *glmmTMB* [52], in which significance of fixed effects was determined using Wald chi-square tests. Variance and 95% confidence intervals for random effects were calculated using the package *stats* [51]. *ggplot2* [53] and *ggeffects* [54] packages were used for plotting. The correlations among early-life measures are shown in electronic supplementary material, figure S3 and table S2. Collinearity between fixed effects was tested for each model using the package *performance* [55]; all variance inflation factors were less than 1.8, suggesting no collinearity between covariates (electronic supplementary material, table S3).

## 3. Results

**Figure 1.**
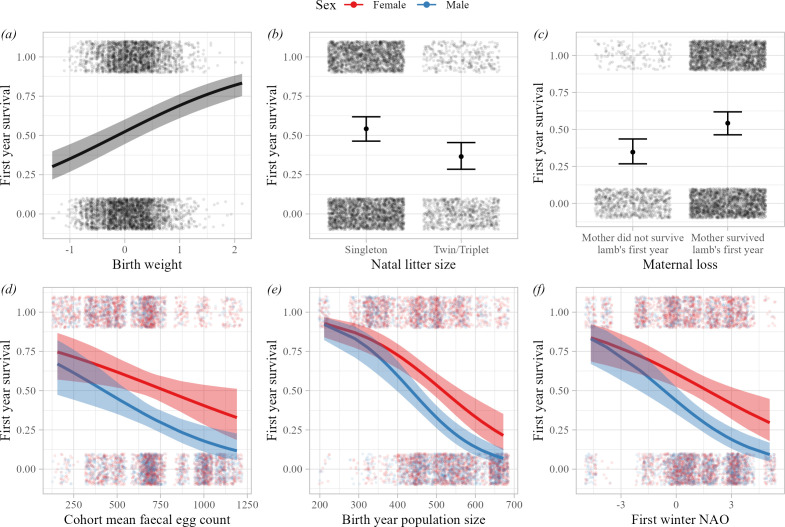
Estimates of the effects of different measures of early-life adversity on first year survival in Soay sheep (*n* = 5074 observations). Plots show independent effects of (*a*) birth weight, (*b*) natal litter size, (*c*) maternal loss during the first year of life, (*d*) cohort mean faecal egg count in year of birth, (*e*) population size in year of birth and (*f*) first winter NAO. Points show the (jittered) raw data. Lines and shading show the predictions and associated 95% CIs from the model in electronic supplementary material, table S4, where all other covariates were held at the mean and factors at the reference level, except maternal loss, which was held at ‘mother survived first year of life’. Plots with coloured lines indicate where the association differed between the sexes; females shown in red, males in blue.

All our measures of early-life adversity were independently associated with first year survival (electronic supplementary material, table S4; figure 1). Twins and individuals who lost their mothers during their first year were less likely to survive their first year of life, as were lambs who were born lighter (maternal loss: estimate = 0.803 ± 0.124 S.E., *p* < 0.001, birth weight: estimate = 0.322 ± 0.046 S.E., *p* < 0.001; electronic supplementary material, table S4; figure 1*a–c*). Those born in years with higher population sizes were also less likely to survive their first year, and this association was stronger for male than female lambs (birth year population size: estimate = −0.925 ± 0.179 S.E., *p* < 0.001, sex-by-population size interaction: estimate = −0.291 ± 0.109 S.E., *p* = 0.007; electronic supplementary material, table S4; figure 1*e*). Lambs who experienced harsh weather conditions during their first winter (high NAO values) had lower survival probabilities, and this association was again stronger for male than female lambs (first winter NAO: estimate = −0.552 ± 0.155 S.E., *p* < 0.001, sex-by-first winter NAO interaction: estimate = −0.301 ± 0.084 S.E., *p* < 0.001; electronic supplementary material, table S4; figure 1*f*). Similarly, lambs in cohorts with higher mean faecal egg counts were less likely to survive to the following spring, and the association was stronger for males (cohort mean faecal egg count: estimate = −0.454 ± 0.184 S.E., *p* = 0.014, sex-by- cohort mean faecal egg count interaction: estimate = −0.237 ± 0.100 S.E., *p* = 0.0.18; electronic supplementary material, table S4; figure 1*d*). There was no evidence that the effect of first winter NAO on first year survival depended on either population size (estimate = 0.059 ± 0.139 S.E., *p* = 0.670; electronic supplementary material, table S5) or birth weight (estimate = 0.005 ± 0.040 S.E., *p* = 0.896; electronic supplementary material, table S5). Birth year explained around 30% more variation in first year survival than maternal identity after accounting for the fixed effects (electronic supplementary material, table S4).

**Figure 2.**
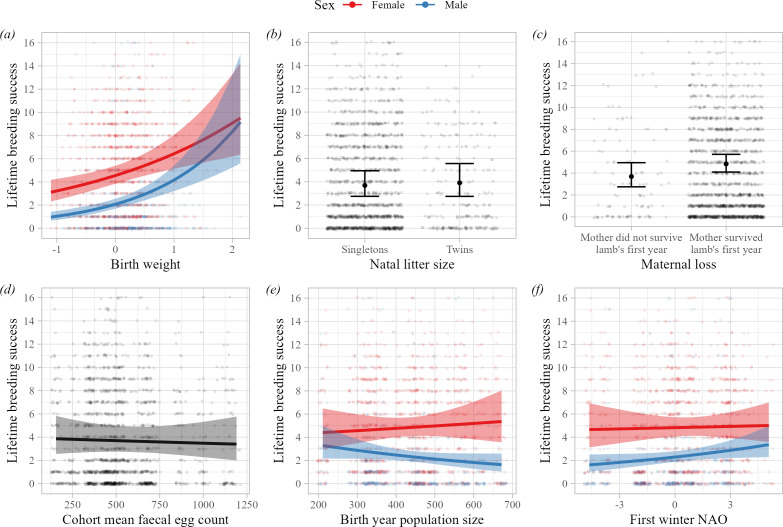
Estimates of the independent effects of (*a*) birth weight, (*b*) natal litter size, (*c*) maternal loss during the first year of life, (*d*) cohort mean faecal egg count in year of birth, (*e*) population size in year of birth and (*f*) first winter NAO on lifetime breeding success in Soay sheep that survived beyond the first year of life (*n* = 1721 observations). Points show the (jittered) raw data. *y*-axes are limited to LBS < 17 for clarity (representing more than 97% of observations). Lines and shading show the predictions and associated 95% CIs from the model in table 1*a*, where all other covariates were held at the mean and factors at the reference level, except maternal loss, which was held at ‘mother survived first year of life’. Plots with coloured lines indicate where the association differed between the sexes; females shown in red, males in blue. The effect of birth weight was statistically significant, as were the effects of population size in year of birth and first winter NAO in males.

The lifetime breeding success (LBS) of individuals that survived beyond their first year was associated with measures of early-life adversity, sometimes in sex-specific ways (table 1*a*; figure 2). Maternal loss in the first year of life was associated with reduced LBS in both sexes (table 1*a*; figure 2*c*). Individuals that were born lighter also had lower LBS, but the effect was stronger in males (table 1*a*; figure 2*a*). There was a significant interaction between sex and population size in the year of birth: males born in high density years had lower LBS, whereas there was no association in females (table 1*a*; figure 2*e*). First winter NAO was also positively associated with LBS in males but not females: males who experienced harsher weather conditions during their first winter performed better as adults (table 1*a*; figure 2*f*). Natal litter size and cohort mean faecal egg count were not significantly associated with LBS in adults (table 1*a*; figure 2*b*,*d*). Once the fixed effects were accounted for, birth cohort explained around 60% more variation in adult lifetime breeding success than maternal identity (table 1*a*).

**Table 1.** Fixed and random effect estimates from negative binomial GLMMs of (*a*) lifetime breeding success, (*b*) longevity and (*e*) male annual offspring number and binomial GLMMs of (*c*) annual breeding probability and (*d*) annual twinning probability in Soay sheep that survived beyond the first year of life. Statistically significant fixed effects are highlighted in bold. The reference levels for the fixed factors were: sex (female), natal litter size (singleton) and maternal loss (mother died in first year of life). Where the effect is included in an interaction, the first-order term shows the effect in females and the interaction term shows the difference in the effect in males compared with females. The fixed covariates were scaled to mean = 0 and standard deviation = 1. Abbreviations: NAO, North Atlantic Oscillation; FEC, faecal egg count.

	(*a*) lifetime breeding success *n* = 1721			(*b*) longevity *n* = 1721			(*c*) annual breeding probability *n* = 9572			(*d*) annual twinning probability (females only) *n* = 5580			(*e*) annual offspring number (males only) *n* = 884		
fixed effects	estimate	std. error	*p*‐value	estimate	std. error	*p*‐value	estimate	std. error	*p*‐value	estimate	std. error	*p*‐value	estimate	std. error	*p*‐value
intercept	**1.305**	**0.150**	**<0.001**	**1.569**	**0.086**	**<0.001**	**−1.714**	**0.233**	**<0.001**	**−6.177**	**0.419**	**<0.001**	**−0.749**	**0.189**	**<0.001**
sex (male)	**−0.723**	**0.073**	**<0.001**	**−0.740**	**0.037**	**<0.001**	**−2.724**	**0.114**	**<0.001**	—	—	—	—	—	—
age	—	—	—	—	—	—	**1.552**	**0.051**	**<0.001**	**1.126**	**0.097**	**<0.001**	**0.431**	**0.047**	**<0.001**
age^2^	—	—	—	—	—	—	**−0.122**	**0.005**	**<0.001**	**−0.067**	**0.008**	**<0.001**	**−0.021**	**0.005**	**<0.001**
birth weight	**0.159**	**0.044**	**<0.001**	**0.063**	**0.020**	**0.001**	**0.260**	**0.054**	**<0.001**	**0.301**	**0.081**	**<0.001**	**0.125**	**0.038**	**0.001**
natal litter size (twin)	0.059	0.103	0.570	−0.041	0.053	0.441	−0.201	0.152	0.187	**0.702**	**0.246**	**0.004**	0.027	0.116	0.813
maternal loss (mother survived)	**0.271**	**0.136**	**0.046**	0.119	0.071	0.093	**0.488**	**0.190**	**0.010**	−0.331	0.307	0.280	**0.334**	**0.163**	**0.040**
cohort mean FEC	−0.027	0.079	0.732	−0.055	0.053	0.299	−0.056	0.101	0.575	−0.021	0.091	0.821	0.037	0.045	0.412
birth year population size	0.049	0.092	0.593	0.088	0.060	0.144	**−0.301**	**0.111**	**0.007**	−0.115	0.098	0.241	**−0.104**	**0.048**	**0.030**
first winter NAO	0.017	0.079	0.828	0.041	0.053	0.441	−0.086	0.090	0.341	−0.073	0.078	0.348	−0.045	0.037	0.227
sex (male): birth weight	**0.161**	**0.068**	**0.017**	—	—	—	—	—	—	—	—	—	—	—	—
sex (male): cohort mean FEC	—	—	—	—	—	—	**0.283**	**0.126**	**0.025**	—	—	—	—	—	—
sex (male): birth year population size	**−0.222**	**0.069**	**0.001**	—	—	—	**−0.350**	**0.121**	**0.004**	—	—	—	—	—	—
sex (male): first winter NAO	**0.157**	**0.068**	**0.021**	**0.120**	**0.036**	**0.001**	—	—	—	—	—	—	—	—	—
random effects	variance	95% CI		variance	95% CI		variance	95% CI		variance	95% CI		variance	95% CI	
maternal identity	0.209	0.141−0.31		0.011	0.001−0.081		0.324	0.172−0.611		0.965	0.559−1.665		0.020	0.001−0.806	
birth year	0.128	0.061−0.265		0.067	0.035−0.127		0.141	0.060−0.332		0.000	0-Inf		0.000	0-Inf	
identity	—	—		—	—		1.539	1.246−1.900		0.975	0.555−1.714		0.146	0.081−0.263	
measurement year	—	—		—	—		0.321	0.181−0.570		0.163	0.076−0.349		0.028	0.012−0.065	
dispersion parameter for negative binomial model	0.932			4.69			—			—			40.1		

The adult longevity component of LBS was positively associated with birth weight in both sexes (table 1*b*; electronic supplementary material, figure S4a). There was a significant interaction between the effects of sex and first winter NAO; males who experienced harsh winter weather during their first year had longer lifespans, whereas female longevity showed no association with first winter NAO (table 1*b*; electronic supplementary material, figure S4b). There was no significant association between litter size, maternal loss, population size in year of birth or cohort mean faecal egg count and adult longevity (table 1*b*). Year of birth explained six times more variation in longevity than maternal identity (table 1*b*).

Annual breeding probability was positively associated with birth weight in both females and males (table 1*c*; electronic supplementary material, figure S5a). Maternal loss in the first year of life was also significantly associated with reduced adult breeding probability in both sexes (table 1*c*; electronic supplementary material, figure S5b). Although higher population sizes in the year of birth were associated with decreased annual breeding probability for both sexes, the effect of population size was significantly stronger in males than females (table 1*c*; electronic supplementary material, figure S5c). The association between annual breeding success and cohort mean faecal egg count differed between the sexes, but the association was not statistically significantly different from zero in either males or females (estimate in females = −0.056 ± 0.101, *p* = 0.575; estimate in males = 0.227 ± 0.136, *p* = 0.089; table 1*c*). Litter size and first winter NAO were not significantly associated with annual breeding probability (table 1*c*). Annual breeding probability was highly repeatable within individuals, with individual identity explaining a much larger proportion of the variance than breeding year, maternal identity or birth cohort once the fixed effects were accounted for (table 1*c*).

Among females that successfully reproduced, females who were heavier at birth were more likely to produce twins (table 1*d*; electronic supplementary material, figure S6a). Annual twinning probability was also significantly higher in females who were twins themselves (table 1*d*; electronic supplementary material, figure S6b). There was no significant association between maternal loss, population size in year of birth, first winter NAO or cohort mean faecal egg count and annual twinning probability (table 1*d*). There was a positive association between birth weight and offspring number in males that successfully reproduced, with males who were born heavier siring significantly more offspring each year (table 1*e*; electronic supplementary material, figure S7a). Males who lost their mother in their first year of life had fewer offspring annually (table 1*e*; electronic supplementary material, figure S7b). Population size in the year of birth was negatively associated with male offspring number, with males born in high density years siring fewer offspring annually (table 1*e*; electronic supplementary material, figure S7c). Litter size, first winter NAO and cohort mean faecal egg count were not significantly associated with annual male offspring number (table 1*e*). Both individual identity and maternal identity explained a large proportion of variance in female twinning probability, whereas individual identity was a more important determinant of male offspring number than measurement year, maternal identity or birth cohort (table 1*d*,*e*).

The positive associations between birth weight and male lifetime breeding success, breeding probability and male offspring number remained statistically significant when controlling for the effects of mean adult weight, though the effect sizes were somewhat reduced (electronic supplementary material, table S6A,C,E). By contrast, birth weight was no longer positively associated with female lifetime breeding success, longevity or female twinning probability once mean adult weight was accounted for (electronic supplementary material, table S6A,B,D). Once mean adult weight was included, the interaction between sex and population size in the year of birth was no longer significant in the models of LBS and breeding probability, and there was no negative association between population size and male offspring number (electronic supplementary material, table S6A,C,E). The associations between first winter NAO and male LBS and male longevity remained when accounting for mean adult weight (electronic supplementary material, table S6A,B).

## 4. Discussion

Our results add to the growing literature demonstrating that early-life environmental conditions have important, long-term effects on adult fitness in wild vertebrate populations [1,21,56–58]. The detailed, longitudinal data collected over four decades on St Kilda enabled us to dissect the effects of multiple aspects of the early-life environment on adult fitness in both male and female Soay sheep. We found that different aspects of the environment experienced during the first year of life had independent and sometimes contrasting impacts on multiple components of adult fitness, particularly in males (illustrated in electronic supplementary material, figure S8). Birth weight was positively associated with adult lifetime breeding success (LBS) in both sexes, but the effect was stronger in males and acted via effects on both longevity and breeding success. However, the negative effect of population size in the year of birth on adult LBS was only detected in males, and operated through effects on reproductive performance but not longevity [59]. These findings are consistent with so-called ‘silver spoon’ effects: individuals that experienced favourable conditions during development performed better as adults [1,6,58]. By contrast, weather conditions experienced during the first winter were predictive of adult male LBS, but the effect was in the opposite direction. Although wet, windy first winters were associated with increased lamb mortality, those males that survived these harsh winters had higher adult LBS than those that experienced more favourable cold, dry winters. This was driven by an effect on adult longevity, and is consistent with stronger viability selection resulting in a cohort where only the more robust, high quality individuals survive to adulthood [13–15]. These marked differences in the relationships between early-life environment, sex and adult fitness highlight the importance of understanding the detail of the processes linking different aspects of early-life adversity and later health and fitness.

First year survival was independently predicted by multiple components of the early-life environment: twins, lighter born lambs, those that lost their mothers in the first year of life, as well as those experiencing higher cohort mean parasite burdens, higher population densities and harsher winter weather were less likely to survive their first winter, confirming previous findings in this study population [33,35,36,44,60]. The associations with cohort mean faecal egg count, population size and first winter NAO were stronger in male than female lambs (electronic supplementary material, table S4; figure 1*d*,*e,f*). This supports our prediction that males would be more sensitive to early-life conditions owing to their larger size and more rapid growth trajectories [25]. Long-term effects of maternal mortality in the first year of life have not previously been investigated in Soay sheep, although other vertebrate studies have shown that ‘orphaning’ impacts offspring survival and later fitness [24,27,61,62]. Although most lambs are weaned by late summer, long before most maternal mortality occurs, they remain closely associated with their mothers for some time, and this effect could reflect social or physiological costs of maternal mortality for the lamb. However, this could also be driven by other environmental factors jointly shaping mother and offspring survival not captured by other early-life variables in our models. Previous studies have documented a relationship between lamb survival and maternal age, with lamb survival lowest in the final year of a female’s life, which is consistent with the relationship observed here [35]. The weak but significant relationship between maternal loss in early life and adult lifetime breeding success (figure 2*c*; table 1*b*) suggests there may be long-term effects of orphaning in this system and merits further, in-depth study.

Birth weight was positively associated with multiple components of fitness in both male and female sheep that survived beyond their first winter (figures 2*a*; electronic supplementary material, figures S4a, S5a, S6a and S7a). Birth weight has been shown to affect later-life reproduction and survival in natural, domestic and laboratory populations [29,63,64], consistent with ‘silver spoon’ effects of early environment. Unlike our cohort-level environmental variables, birth weight varies within years and reflects variation among individual mothers in their ability to care for, and invest in, their offspring [36]. In Soay sheep, birth weight is influenced by maternal genetic and environmental effects, and is positively associated with lamb weight in summer and adult weight [31,34,45,65,66]. Birth weight was predictive of male LBS independent of mean adult weight, suggesting that the effects are not solely due to heavier neonate lambs growing faster and developing into heavier, higher condition adults (electronic supplementary material, table S6A). The observed effects of birth weight may reflect long-term impacts on development and physiological function of maternal investment during both gestation and lactation, as mothers in better condition are likely to invest more during both phases of development. They could also reflect fine-scale spatial variation in the mother’s environment as home range quality has been shown to influence fitness, and this is shared with the offspring over the first year of life [67]. The effect of birth weight on LBS was stronger in males than females, in line with our predictions and previous studies of wild mammals, which suggest that males gain greater fitness benefits from being born heavier than females [29]. Male growth trajectories are much steeper than those for females in the first year of life; males born into environments with reduced food resources may be unable to reach high enough body size to successfully compete against other males in the first few years of life, or may allocate more resources to growth than reproduction in order to catch up [68–71]. Although female LBS was associated with birth weight, this association was driven by the fact that heavier born females were heavier as adults. By contrast, the associations between birth weight and multiple components of fitness were independent of adult weight in males, suggesting that early nutritional deficits may have more fundamental consequences for males. Our findings highlight the sensitivity of the earliest stages of development and demonstrate that compensatory growth is not always sufficient to overcome nutritional stress experienced very early in life.

First winter NAO had an unexpected positive effect on the longevity of males that survived challenging conditions during their first winter, which translated into an increase in male adult LBS (figure 2*f*; electronic supplementary material, figure S4b). Winter NAO is thought to predict fitness in the sheep because it captures the thermoregulatory challenges associated with the frequent and severe winter storms that kill already food-limited sheep on St Kilda [33,44,72]. It is therefore an indicator of a direct environmental driver of lamb mortality in late winter. Our results are consistent with harsh conditions leading to stronger viability selection, leaving only the most robust individuals in a cohort. However, we did not detect a differential impact of first winter NAO depending on birth weight, suggesting that this heterogeneity within a cohort is not straightforward to capture. Long-term studies in other systems have shown that strong early-life viability selection can produce adult cohorts with increased longevity [13–15,23]. These effects may manifest through longevity rather than reproduction if they operate through differences in average physiological condition and resilience among birth cohorts, which also drive differences in adult survival. The effects of first winter NAO were sex-specific; there were no significant effects of first winter weather on female adult fitness traits (figure 2*f*; electronic supplementary material, figure S4b). This fits with both the general prediction that viability selection is stronger in males in polygynous systems [73,74], and the finding that male lamb survival is more strongly influenced by winter NAO in this population (figure 1*f*).

We also found long-term, sex-specific effects of population size in the year of birth on adult LBS (figure 2*e*). Population size did not influence adult longevity, instead depressing annual breeding probability in both sexes, and the number of offspring sired in males (electronic supplementary material, figures S5c and S7c). These ‘silver spoon’ effects contrast with the findings for first winter NAO. Their opposing effects on adult LBS likely reflects the fact that these two early-life predictors influence adult fitness via different processes, despite both being measured at the cohort level. Annual population size shapes the development and demography of the sheep via effects on resource availability and density dependence. Rather than reflecting a direct mortality risk in winter, it captures the level of competition for food and mates across the study area over the entire year. Other studies have shown similar negative effects of high early-life population density on later fitness in wild vertebrates [7,75,76]. These are typically attributed to reduced resource availability and nutritional stress in early life having a negative impact on development and thus adult condition and fitness [23,56]. On St Kilda, this effect may be most acutely felt during the lamb’s first autumn and winter when the animals are growing fast, and plant food resources are at their most limited. This could be somewhat independent of birth weight effects, if these are more reflective of maternal investment and conditions experienced through the previous winter and spring. High sheep numbers, reflecting high competition for limited food late in the year, should negatively impact growth rates and, potentially adult size and condition. This would fit with the finding that early population size effects are stronger for males, for whom growth is more rapid from the first year onwards, and adult fecundity is more strongly size- and condition-dependent compared with females [31,59]. Accounting for mean adult body weight rendered the sex-by-population size interaction non-significant in the models of LBS and annual breeding probability, and the effect of population size non-significant in the model of male offspring number (electronic supplementary material, table S6). This supports the hypothesis that first year population size effects on reproductive performance are mediated by resource limitation and competition impacting growth and ultimately adult body size and condition.

Some studies have suggested that the effects of the early environment may differ depending on conditions experienced during adulthood [4,56,77]. In particular, there may be correlations between the early and adult environments. For example, population size in year of birth may influence the levels of sexual competition males experience as adults. Annual population density is known to be negatively associated with reproductive success for males of all ages in this system, and males may have higher lifetime breeding success if they experience less mate competition as juveniles. The predictive adaptive response hypothesis, on the other hand, proposes that individuals perform best when the adult environment matches their natal environment [78]. For example, individuals in cohorts with high parasite exposure may invest heavily in immunity meaning they perform better as adults when parasite burdens are high but may lose out to competitors who invest more in reproduction when prevalence is low. We did not test explicitly whether the impacts of early-life adversity were dependent on the adult environment primarily because of the large number of environmental variables in our analyses, particularly considering the potential for interactions between early and adult conditions. We did include cohort as a random intercept term in all our models to account for the fact that individuals born in the same year will encounter similar environments as adults. However, future studies considering the potential interactions between developmental and adult environments at both the individual and cohort levels may shed further light on the relative importance of silver spoon versus viability selection for shaping adult performance in wild systems.

Early-life environmental conditions influence the developmental trajectories, behaviour, life history and ultimately fitness of individuals, and can impose strong viability selection on cohorts, in natural populations [1,13,79]. Although there is abundant evidence to support the existence and ecological and evolutionary significance of such effects, natural populations experience a range of different environmental challenges which may not all have the same effect within a given population. Human and primate studies have sought to combine and simplify multiple early-life environmental factors into single metrics of cumulative early-life adversity [17–19], although a recent study of wild hyenas showed that this approach reduced the power to explain variation in adult fitness [21]. Our detailed analysis of the long-term fitness effects of well-understood early environmental measures demonstrates that different aspects of the early environment may show contrasting associations with adult fitness depending on whether they operate through increased viability selection or silver spoon effects, and whether they are measured at the cohort or individual level. We also found that the conditions experienced during development can impact multiple components of fitness in sex-specific ways, further highlighting the complexity of early-life adversity effects. Our findings emphasize both the importance of long-term, individual-based studies for our understanding of variation in fitness and demography in wild animal systems [80] and the need for a more detailed understanding of how specific environmental pressures in early life shape development, viability selection and adult reproduction, health and survival.

## Data Availability

Data and accompanying description are available from the Dryad Digital Repository [81]. Supplementary material is available online [82].
